# Patterns of presentation and early treatment outcomes of anterior cruciate ligament injury at the National Orthopaedic Hospital, Lagos, Nigeria: a retrospective cross-sectional study

**DOI:** 10.11604/pamj.2022.41.315.29153

**Published:** 2022-04-20

**Authors:** Oladimeji Ranti Babalola, Wahab Egberongbe, Kehinde Sunday Oluwadiya

**Affiliations:** 1 Division of Arthroscopy and Sports Medicine, Department of Orthopaedics and Trauma, National Orthopaedic Hospital, Lagos State, Nigeria,; 2 Department of Orthopaedics, Ekiti State University Teaching Hospital, Ekiti State, Nigeria

**Keywords:** Prevalence, anterior cruciate ligament, gender, sports, aetiology, population, males, females, mechanism, injury

## Abstract

The purpose of this study was to determine the patterns of presentation and early treatment outcomes of anterior cruciate ligament tears at the National Orthopaedic Hospital, Lagos, Nigeria. This was a retrospective cross-sectional study in which the details of all anterior cruciate ligament injuries seen from January 2014 to December 2018 in our facility were noted. The bio-demographic details of the patients were noted as well as the side of the injury, mechanism of injury, type of sporting activity patient was engaged in at the time of injury and the early outcome of treatment of the patients were noted. A total of 19,707 new orthopaedic and musculoskeletal trauma cases were seen in the period. The overall hospital period prevalence rate of anterior cruciate ligament injury in the period was 3.6 per 1000 patients with a gender-specific prevalence rates of 5.7 per 1000 and 1.6 per 1000 for male and female patients respectively. The mean time between injury and presentation was 16 (±21) months with a range of 1-120 months. The commonest aetiology of anterior cruciate ligament injury was non-contact injury during sporting activities. There was a greater involvement of the young and active population in this injury. Sports and road traffic crash related injuries were the commonest injury aetiology in our environment. The early treatment outcomes revealed a significant improvement of the post-operative functional knee scores over the pre-operative functional knee scores.

## Introduction

Anterior cruciate ligament (ACL) injuries are one of the common injuries around the knee [1]. Sporting activities such as skiing, rugby and soccer have been noted to have a high incidence of this injury [2]. The literature has shown that this injury may also vary by gender and response to injury-reduction training programs [3-6]. On a sport specific basis, sports such as gymnastics, in-door obstacle course and basketball have been noted to have a higher incidence of ACL injuries in females than in males [3]. Anterior cruciate ligament injuries tend to occur as part of a spectrum of knee injuries that may include meniscal, chondral or a second ligament injury.

Previous studies have described the epidemiology of ACL injuries from other regions outside of Nigeria and West Africa [7, 8]. However, our search did not yield any study relating to the data on this injury from Nigeria or the West African region. This is important as a record of this nature has been found to be very useful in the prevention of this injury. The purpose of this study was to determine the patterns of presentation and the early treatment outcomes following treatment of ACL tears presenting at the National Orthopaedic Hospital, Lagos, Nigeria.

## Methods

**Study design and setting:** this was a retrospective cross-sectional study aimed at determining the injury patterns and early treatment outcomes of anterior cruciate ligament injury in patients presenting at the out-patient clinic and emergency room of the National Orthopaedic Hospital, Igbobi, Lagos, Nigeria (NOHI). NOHI is a regional orthopaedic center in the country for the treatment of musculoskeletal diseases. The hospital is focused primarily on the care of orthopaedic disorders and is a regional referral center in the southern region of Nigeria for ACL injury treatment.

**Study population:** the details of all patients diagnosed with anterior cruciate ligament injury between January 2014 to December 2018 and whose details had been entered into a registry in the arthroscopy unit of the Hospital were reviewed. These were patients who were clinically diagnosed with the injury and had it confirmed on magnetic resonance imaging, and subsequently had primary arthroscopic reconstruction of the torn anterior cruciate ligament. Patients with a re-rupture of a previously constructed ACL injury were excluded from the study.

**Data collection:** the bio-demographic details of the patients such as the age, sex and anthropometric characteristics were extracted from the records. The record was a registry of patients who had primary ACL reconstruction following ACL rupture. The registry contained details relating to the clinical findings at presentation, type of ACL reconstruction done based on graft choice and the short-term outcome of management of the patients using the international knee documentation committee (IKDC) score. The practice in our institution is to perform an arthroscopic ACL reconstruction using either autogenous tripled semitendinosus graft or quadrupled semitendinosus-gracilis tendon graft.

Also noted was the side of the injury, mechanism of injury and type of sporting activity patient was engaged in when the injury occurred. In addition, the interval between injury and presentation was noted. An ACL injury was considered acute if diagnosed within 6 weeks or less from injury and chronic if diagnosed later than 6 weeks [9]. The pre-operative and six-month post-operative international knee documentation committee (IKDC) scores of the injured knee in the register were also noted as well as the complications reported by the patients within six months of post-operative follow-up care.

**Statistical analysis:** IBM SPSS version 25 (IBM, Chicago, IL, USA) was used to analyse the data. The normality of continuous variables was first determined using Kolmogrov-Smirnov statistics, and the result determined our choice of univariate analysis of the variables. Categorical data were analysed using proportions. Charts and tables were used to present results of univariate and bivariate analysis. We used chi-square test to explore the relationship between categorical data and independent T-test to compare the means of continuous data and a p-value of less than 0.05 was deemed significant.

**Ethical considerations:** ethical clearance was obtained from the Institutional Health Research and Ethical Committee, (Reference number; OH/90/C/IX).

## Results

**Figure 1:**
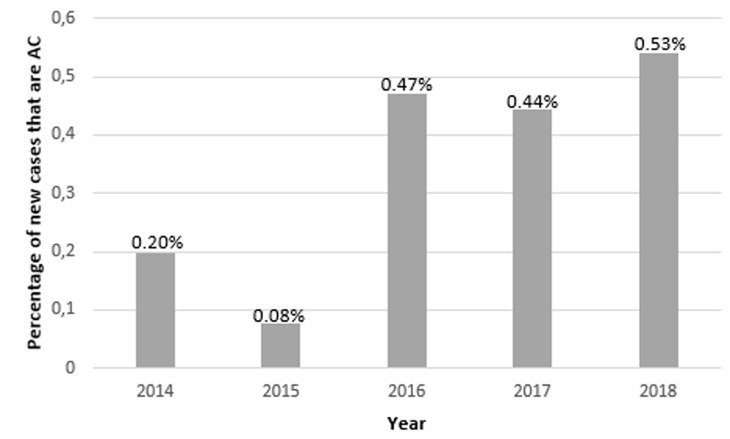
new cases of anterior cruciate ligament injury as a percentage of total number of new cases seen each year

**Figure 2:**
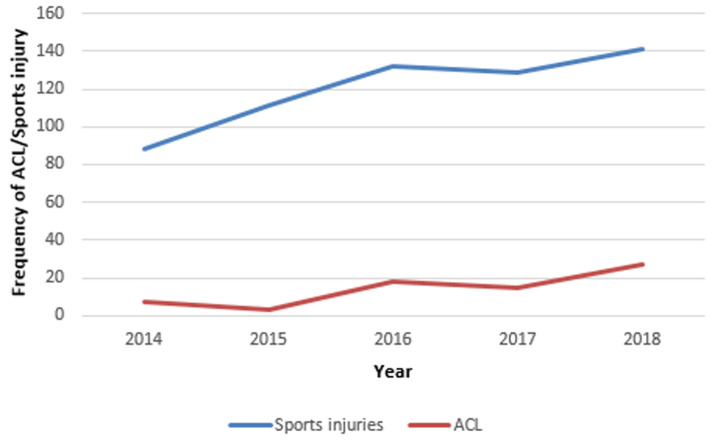
trend of anterior cruciate ligament (ACL) injuries and sports injury, 2014-2018

**Frequency and trends of ACL over time:** a total of 19,707 new orthopaedic and musculoskeletal trauma patients were seen in the emergency and orthopaedic out-patient departments of the hospital during the study period. Of these, 601 cases (3.1%) were sports-related injuries, and 70 (0.36%) of the total number of new cases were specifically anterior cruciate ligament injuries. Of the 19,707 new orthopaedics and musculoskeletal trauma cases, 9335 (47%) were males and 10,372 (53%) were females with a sex ratio of 1: 1.1. There were 601 new cases of sports injuries observed over the study period. There were 70 cases of ACL injury. Of the cases of ACL injuries, 52 (74%) were males and 18 (26%) were females, with a sex ratio of 3: 1 (P<0.001). The data gives an overall period prevalence rate of 3.6 per 1000 patients for ACL injury within the study period and a gender-specific prevalence rates of 5.7 per 1000 and 1.6 per 1000 for males and females respectively. The age distribution of the male and female ACL injured patients is as displayed in Table 1. Over the 5-year period, except for a slight dip in 2015, there was a gradual increase in the number of ACL injuries diagnosed (Figure 1, Figure 2).

**Table 1:** frequency distribution of ACL injury among the age groups in the study

Age groups (years)	0-10	11-20	21-30	31-40	41-50	51-60	61-70
Females (f)	0	1	5	7	5	0	0
Males (f)	0	8	23	12	6	1	2
Total	0	9	28	19	11	1	2

Considering the laterality of anterior cruciate ligament injuries; fifty (71%) patients had their injuries in the right knee, while twenty (29%) had the injury in the left knee. There was no statistically significant difference between the side involved in the injury between sports-related and non-sports related ACL injury cases (Table 2). The mean time between injury and presentation was 15 (±21) months. Fifty-five cases (79%) presented as chronic injuries, while 15 (21%) presented as acute injuries. Based on injury mechanism; 35 (50%) of the cases were related to sporting activities (Table 2). This represents 6 % of sports related injuries seen within the study period.

**Table 2:** comparing anterior cruciate ligament injury between those involved in sporting and non-sporting activities at the time of injury on gender basis and laterality basis

		Sports (%)	Non-Sports (%)	Total	P-value
**Gender**	Male	31 (60.0)	21 (40.0)	52	0.03
	Female	4 (22.2)	14 (77.8)	18	
	Total	33 (47.1)	37 (52.9)	70	
**Left/Right**	Right	24 (54.5)	20 (46.5)	44	0.14
	Left	9 (34.6)	17 (65.4)	26	
	Total	33 (47.1)	37 (52.9)	70	

**Risk factors and mechanisms of the injury:** twenty-three (33%) of the other cases of ACL injuries resulted from road traffic crashes, 8(11%) cases from falls from heights, three (4%) from occupational accidents and one (2%) from domestic fall. Of the cases due to sporting injuries, 29 (82%) occurred in soccer matches, 3 (9%) during high jumps, 2 (6%) while sprinting, and 1 (3%) during an American football match. Nineteen (66%) of the cases resulting from soccer were due to non-contact injuries, while 10 (34%) were due to contact injuries.

**Clinical presentation:** the commonest injury combination was an ACL injury occurring together with both a medial meniscal tear and a medial collateral ligament injury (30%) and an ACL injury occurring with both a medial meniscal injury and a lateral meniscal tear (20%). Isolated ACL injury was observed in only 25% of the cases in our cohort. There were only five cases of associated posterior cruciate ligament injury, with 4 (80%) of these cases due to road traffic crashes. As Table 2 shows, a significantly higher proportion of males had sports-related ACL injuries compared to females (P=0.03). Sports (60%) was the commonest cause of injuries in males, and road traffic crashes (41%) were the commonest cause in females.

**Management and outcome:** all patients included in the study had primary arthroscopic anterior cruciate ligament reconstruction using autogenous semitendinosus graft. The short-term functional outcome was determined by comparing the pre-operative international knee documentation committee (IKDC) score with the IKDC score at six months. There was a statistically significant (p=0.02) improvement of IKDC score at 6 months (65.90±9.30) over the pre-operative values (49.65±12.17). Three cases (4%) of knee sepsis were recorded in our series. Other complications noted were spinal headaches in 8 (11%) and 8 (11%) cases of severe post-operative knee pain. The cases of knee sepsis were managed with arthroscopic joint lavage with gentamycin-containing normal saline. One patient of the three cases of knee sepsis needed to have a distal femoral knee replacement on account of persistent post-operative knee sepsis.

## Discussion

The purpose of this study was to determine the patterns of presentation and early treatment outcomes of ACL tears presenting at the National Orthopaedic Hospital, Lagos, Nigeria. Our study revealed a period prevalence of 3.6 per 1000 patients for ACL injury in the institution of study. A greater involvement of the young and active population in this injury was observed in our study. Sports and road traffic crash related injuries were the commonest injury aetiology in our environment. The short-term functional outcome of care revealed a significant improvement over the pre-operative functional knee scores. The complications observed in following arthroscopic ACL reconstruction in our series were spinal headaches, severe knee pain and knee sepsis.

Anterior cruciate ligament injuries are known to have debilitating effects with long term consequences that may affect the quality of life of the individual [10, 11]. Their short- and long-term impacts on knee function have been likened to the impact of a cerebrovascular accident on body function [12]. One of the strengths of our study is the fact that the prevalence of this injury recorded in our cohort was expressed as a function of the total number of new cases seen over the period of the study, as against being a function of the number of play hours as used in previous studies. This is because very few of the patients participated in professional sports and had no record of their hours of exposure to sporting activities.

Also, the aetiology of the injury in this series was varied with sports injury and road traffic crash being the commonest mechanism of injury. There was an unexplained dip in the number of ACL injuries that was not mirrored in the number of sports injuries. However, the number of ACL injuries in 2016 onward were more than double the number of injuries presenting in 2014. This may be indicative of an improved clinical acumen of surgeons and residents in clinical diagnoses as well as the increasing use of Magnetic Resonance Imaging in helping to confirm clinical diagnoses. The mean age observed in the study lends further support to the fact that anterior cruciate ligament injury is, indeed a disease of the young and active [13]. Females have been observed to have a 3-10 times risk of ACL injury compared to males when exposed to the same sporting activity [13, 14].

Our study however, revealed a relatively higher period prevalence of ACL injuries in males compared to females. This may be related to the relatively lower female participation in recreational and professional sporting activities in our study environment within the study period as most of the cases of ACL injuries in this study resulted from sporting activities [15, 16]. Sporting activities have been noted to be the commonest cause of anterior cruciate ligament injuries [17, 18]. The higher risk gender poses on females for ACL injuries have been noted to decline significantly from adolescence to adulthood [19, 20]. The mean values of the age at presentation in our study was well above the adolescent age bracket and may not have been a strong factor in the aetiology of the injuries in our cohort. Most of the injuries in our study were due to relatively low energy transfer injuries that is experienced during sporting activities as against the high energy transfer of road traffic crashes and falls from height. Most of the sporting injuries were non-contact injuries during soccer. This is a sporting activity that involves a lot of under-cutting and pivoting movements both of which have been observed to increase the risk of non-contact injuries.

However, our value for non-contact injuries is lower than that observed in previous studies [15, 21]. This may be related to the smaller sample size of our study. Anterior cruciate ligament injuries are rarely isolated injuries. The commonest associated ligament injury observed in our study was a medial collateral ligament injury. This is similar in the findings in a previous study relating to the frequency of medial collateral ligament injuries [22]. It is easy to conceptualize the involvement of the medial collateral ligament injury in the classic “position of no return” mechanism of non-contact ACL injury in which the hip on the injured side is initially internal rotated and adducted, and the knee goes into valgus, and tibia into external rotation on a pronated, externally rotated foot [23]. The delay in presentation observed among the patients in our study may be attributed to a delay in accurate diagnosis, the poor health seeking behaviour prevalent in our study environment and poor health insurance coverage [24, 25]. The delay in presentation was much longer than the average duration of one week reported in the study by Arastu *et al*. [26]. Majority of patients who had their surgery within the study period had to pay out of pocket. Most patients are usually not financially prepared for the high cost of surgery and rehabilitation associated with arthroscopic ACL reconstruction. A delayed presentation in ACL injuries have been observed to cause more damage to the injured knee and further accelerate the progressive of osteoarthritis in the injured knee [26, 27]. The short-term functional outcome of the management of these patients revealed a significant increase in the knee scores over the pre-operative values. This reflects the improvement in the stability of the knee the operative intervention afforded the patients.

Post-spinal headache and significant knee pain were the commonest post-operative complications observed in our study. The spinal headaches may be explained by the fact that the hospital uses a 25 gauge bevelled spinal needle for regional anaesthesia and the cases of spinal headaches were cases where multiple attempts were made at the securing the regional anaesthesia. The use of atraumatic spinal needles with a gauge of 24-27G and a diamond-shaped atraumatic tip and with fewer lumbar puncture attempts has been observed to significantly lower the risk of spinal headache [28]. The severe knee pain observed were related managed with a review in the post-operative analgesia in the cases involved to allow for a better pain control with improvement observed in the pain symptoms. Three cases of knee sepsis were observed in our study were managed with arthroscopic knee lavage and addition of gentamycin to the last pint of irrigation fluid. One of the patients with knee sepsis who had persistent knee infection was offered distal femoral replacement after initial insertion of an antibiotic spacer to replace the resected distal femur to control infection before insertion of the prosthesis. The limitations of our study include its retrospective nature and the relatively small sample size and the fact that it is a single centre study. This may imply that some cases occurring in the community may not have been captured.

## Conclusion

Our study reveals a greater involvement of the young and active population in ACL injury. Sports and road traffic crash related injuries are the commonest injury aetiology of ACL tear in our environment. The early treatment outcomes of care revealed a significant improvement over the pre-operative functional knee scores.

### What is known about this topic

- Anterior cruciate ligament injuries are well documented in the western world;
- Sports injuries represents a distinctly important and common aetiology of the injury;
- There is a higher risk of this injury in females compared have been observed compared to males.

### What this study adds

- The study describes the pattern of presentation and early treatment outcome in a center in the West African region;
- The study noted a significant contribution of road traffic injury to the aetiology of anterior cruciate ligament tears;
- The study noted a relatively lower injury rate in females as compared to males in the study environment.
